# The impact of transient combination antiretroviral treatment in early HIV infection on viral suppression and immunologic response in later treatment

**DOI:** 10.1097/QAD.0000000000000991

**Published:** 2016-03-07

**Authors:** Nikos Pantazis, Giota Touloumi, Laurence Meyer, Ashley Olson, Dominique Costagliola, Anthony D. Kelleher, Irja Lutsar, Marie-Laure Chaix, Martin Fisher, Santiago Moreno, Kholoud Porter

**Affiliations:** a Department of Hygiene, Epidemiology and Medical Statistics, Athens University Medical School, Athens, Greece; b Faculte de Medecine Paris Sud; c INSERM U1018, Centre de recherche en Epidemiologie et Sante des Populations, Universite Paris Sud, Le Kremlin Bicetre, France; d Medical Research Council Clinical Trials Unit, University College London, London, UK; e INSERM; f Sorbonne Universités, UPMC Univ Paris 06, UMRS 1136, Institut Pierre Louis d’Epidémiologie et de Santé Publique, Paris, France; g Kirby Institute, UNSW, Sydney, Australia; h Department of Microbiology, Faculty of Medicine, University of Tartu, Tartu, Estonia; i Laboratoire de Virologie, Hopital Saint Louis, Universite Paris Diderot, INSERM U941, Paris, France; j HIV/GUM Research, Brighton and Sussex University Hospitals NHS Trust, Brighton and Sussex Medical School, Brighton, UK; k Infectious Diseases Department, Hospital Universitario Ramón y Cajal, Instituto Ramón y Cajal de Investigación Sanitaria, Madrid, Spain.

**Keywords:** early/primary HIV infection, immunologic response, transient combination antiretroviral treatment, treatment reinitiation, virologic response

## Abstract

**Objective:**

Effects of transient combination antiretroviral treatment (cART) initiated during early HIV infection (EHI) remain unclear. We investigate whether this intervention affects viral suppression and CD4^+^ cell count increase following its reinitiation in chronic infection (CHI).

**Design:**

Longitudinal observational study.

**Methods:**

We identified adult patients from Concerted Action of Seroconversion to AIDS and Death in Europe who seroconverted after 1/1/2000, had a 12 months or less HIV test interval and initiated cART from naive. We classified individuals as ‘pretreated in EHI’ if treated within 6 months of seroconversion, interrupted for at least 12 weeks, and reinitiated during CHI. Statistical analysis was performed using survival analysis methods and mixed models.

**Results:**

Pretreated and initiated in CHI groups comprised 202 and 4263 individuals, with median follow-up after CHI treatment 4.5 and 3 years, respectively. Both groups had similar virologic response and relapse rates (*P* = 0.585 and *P* = 0.206) but pretreated individuals restarted treatment with higher baseline CD4^+^ cell count (∼80 cells/μl; *P* < 0.001) and retained significantly higher CD4^+^ cell count for more than 3 years after treatment (re)initiation. Assuming common baseline CD4^+^ cell count, differences in CD4^+^ cell count slopes were nonsignificant. Immunovirologic response to CHI treatment was not associated with timing or duration of the transient treatment.

**Conclusion:**

Although treatment interruptions are not recommended, stopping cART initiated in EHI does not seem to reduce the chance of a successful outcome of treatment in CHI.

## Introduction

For the vast majority of untreated HIV-positive individuals, the course of disease is characterized by sustained viral replication and progressive CD4^+^ T-cell depletion [1]. The ability of combination antiretroviral treatment (cART) to suppress viral load and boost the immune system is well established [2]. Unfortunately, cART cannot eradicate HIV [3], and treatment interruption is usually followed by rapid viral rebound, CD4^+^ T-cells loss [4], and increased risk of morbidity and mortality [5]. Commitment to cART is, therefore, lifelong.

It has been shown that immune response during acute HIV-1 infection plays an important role on subsequent disease progression [6], and that cART administered during that period may preserve HIV-1–specific immune response [7–9], reduce viral diversity and reservoir size [10,11], and stimulate immune restoration [12,13]. Thus, it was hypothesized that transient cART during early HIV infection (EHI) could lead to prolonged control of viral replication, even after its interruption.

Several studies have focused on the effect of transient cART initiated soon after seroconversion [14–25]. However, despite discrepancies in findings, it is clear that, even in the best case scenario, a small proportion of these early-treated individuals maintain long-term viral suppression after cART cessation.

International guidelines, specifically regarding treatment of acute or early HIV-1 infection, remain unclear. United States Department of Health and Human Services guidelines recommend that cART should be offered to all patients with EHI [26]. European AIDS Clinical Society guidelines suggest that treatment should be considered and actively discussed [27]. WHO guidelines do not contain any relevant recommendations for acute/early infection [28].

In practice, a substantial number of HIV-positive persons diagnosed soon after seroconversion initiate cART immediately, even without having a low CD4^+^ cell count (i.e. with >500 CD4^+^ cells/μl), or AIDS-related conditions. Despite the contraindications for treatment interruption, many do stop, and the vast majority loses the virologic and immunologic advantages gained while on cART. These individuals will most likely have to reinitiate treatment later, during chronic infection (CHI). Whether the response to cART reinitiated in CHI for these individuals with prior transient cART experience in EHI differs from that of those initiating in CHI, has not been sufficiently evaluated.

The main objective of this study was to assess if transient cART, administered during EHI, has any effect on response to cART reinitiated during CHI. Additionally, differential effects associated with the timing and duration of transient cART initiated soon after seroconversion was investigated.

## Methods

CASCADE (Concerted Action of Seroconversion to AIDS and Death in Europe – http://www.cascade-collaboration.org/) is a collaboration of 28 cohorts of individuals with well-estimated dates of HIV seroconversion [29]. We used data pooled in September 2014 within EuroCoord (www.EuroCoord.net). All collaborating cohorts received approval from their regulatory or national ethics review boards.

In CASCADE, seroconversion dates are estimated as the midpoint between the last documented negative and first-positive HIV antibody test dates for the majority of participants (87.8%) with the interval between these tests being 3 years or less. For the remaining individuals, seroconversion dates are estimated through laboratory methods (PCR positivity in the absence of HIV antibodies or antigen positivity with fewer than four bands on western blot; 10.1%), or as the date of seroconversion illness with both an earlier negative and a later positive HIV test performed within 3 years or less (2.1%).

Eligible individuals were those who seroconverted in or after 2000, were at least 15 years old, had their seroconversion date determined through the midpoint (test interval ≤12 months) or laboratory methods, and had initiated cART from naive. Individuals followed-up in African cohorts and those who started cART in EHI with AIDS or with a prior CD4^+^ cell count of less than 350 cells/μl were excluded. Individuals, who started cART during EHI were eligible only if they remained on treatment for at least 12 weeks and less than 2 years, interrupted all cART drugs for at least 12 weeks and reinitiated later during CHI. Those who received cART during CHI, for less than 8 weeks or did not have CD4^+^ cell count and viral load measurements at baseline (i.e. cART initiation in CHI) and while on treatment, were excluded.

We defined EHI as the first 6 months after seroconversion [26] and CHI thereafter. Virological response was defined as two consecutive viral load measurements less than 50 copies/ml. Virological relapse was defined as having two consecutive viral load measurements at least 50 copies/ml or just one at least 100 copies/ml, after virological response. cART was defined as a regimen containing at least three antiretroviral agents from at least two different classes or combinations of at least three nucleoside reverse transcriptase inhibitors with one of them being abacavir or tenofovir.

### Statistical methods

Virologic response and relapse rates were analyzed using survival analysis methods for interval censored data (nonparametric maximum likelihood estimator of the survivor function and accelerated failure time models [30]) as the exact times of these events were only known to lie between the times of two successive viral load measurements. CD4^+^ cell count dynamics after (re)initiation of cART during CHI were analyzed using a piecewise linear mixed model with different slopes for the first 3 months, months 3–30 and after the 30th month of treatment after a square root transformation. A postestimation adjustment was used to compare the two groups in terms of CD4^+^ cell count evolution, assuming a common baseline CD4^+^ cell count [31]. Age, sex, risk group, ethnic group, year of seroconversion, acute infection, previous AIDS, seroconversion determination method, year of treatment initiation, and baseline marker levels were considered as potential confounders.

## Results

Of 31 482 seroconverters in CASCADE, 24 482 did not meet the inclusion criteria (Figure S1). The final study population consisted of 4465 individuals, of whom 202 received transient cART during EHI and restarted cART in CHI (‘Pretreated in EHI’ group). The remaining 4263 individuals who initiated cART in CHI will be referred to as the ‘Previously untreated’ group.

Demographic and clinical characteristics are presented in Table 1. The two groups were comparable in terms of sex, risk group, type of CHI-cART regimen, and CHI-cART baseline viral load distributions. Those pretreated in EHI tended to be slightly older, acquired HIV in earlier years, and had higher viral load and CD4^+^ cell count close to seroconversion, higher CD4^+^ cell count at the start of CHI-cART, and longer post CHI-cART follow-up. CHI-cART regimens for both groups were almost equally based on nonnucleoside reverse transcriptase inhibitor (NNRTIs) or boosted protease inhibitors (PIs), with only a small proportion starting a triple NRTI or fusion/maturation inhibitor-based regimen. Finally, the time of cART (re)initiation in CHI, relative to seroconversion, was longer in those pretreated in EHI compared with those previously untreated.

**Table 1:** Demographic and clinical characteristics of HIV-1 seroconverters treated in chronic infection by whether they had initiated combination antiretroviral treatment within 6 months of seroconversion (pretreated in early HIV infection) or not (not-pretreated in early HIV infection).

	Group			
	Pretreated in EHI (*n* = 202)	Not-pretreated in EHI (*n* = 4263)	Overall (*N* = 4465)	*P* value
Female sex	21 (10.4)	533 (12.5)	554 (12.4)	0.375
Risk group				0.140
MSM	154 (76.2)	3193 (74.9)	3347 (75.0)	
IDU	1 (0.5)	135 (3.2)	136 (3.0)	
MSW	38 (18.8)	798 (18.7)	836 (18.7)	
Other-unknown	9 (4.5)	137 (3.2)	146 (3.3)	
Ethnic group				<0.001
White	131 (64.9)	1668 (39.1)	1799 (40.3)	
Black	7 (3.5)	103 (2.4)	110 (2.5)	
Hispanic	0 (0.0)	27 (0.6)	27 (0.6)	
Asian	4 (2.0)	41 (1.0)	45 (1.0)	
Mixed/other	0 (0.0)	21 (0.5)	21 (0.5)	
Unknown	60 (29.7)	2403 (56.4)	2463 (55.2)	
Seroconversion determination and HIV test interval				<0.001
Midpoint (>30 days)	69 (34.2)	3230 (75.8)	3299 (73.9)	
Midpoint (≤30 days)	14 (6.9)	121 (2.8)	135 (3.0)	
Laboratory evidence	109 (54.0)	763 (17.9)	872 (19.5)	
Seroconversion illness	10 (5.0)	149 (3.5)	159 (3.6)	
Type of EHI-cART				
NNRTI	42 (20.8)			
Boosted PI	141 (69.8)			
Unboosted PI	12 (5.9)			
Other	7 (3.5)			
AIDS before CHI-cART	11 (5.4)	153 (3.6)	164 (3.7)	0.170
Type of CHI-cART				0.377
NNRTI	87 (43.1)	1994 (46.8)	2081 (46.6)	
Boosted PI	100 (49.5)	1875 (44.0)	1975 (44.2)	
Unboosted PI	6 (3.0)	120 (2.8)	126 (2.8)	−
Other	9 (4.5)	274 (6.4)	283 (6.3)	
Age at seroconversion (years)	35.4 (30.1, 42.0)	33.6 (27.5, 40.9)	33.7 (27.6, 40.9)	0.009
Year of seroconversion	2003 (2002, 2004)	2006 (2003, 2008)	2006 (2003, 2008)	<0.001
Age at CHI-cART (years)	39.7 (33.9, 46.4)	36.4 (30.2, 43.6)	36.5 (30.3, 43.7)	<0.001
Months from seroconversion to EHI-cART	0.6 (0.3, 2.3)			
EHI-cART duration (months)	11.0 (5.7, 14.6)		11.0 (5.7, 14.6)	
TI duration of pretreated (months)	35.4 (17.8, 57.2)		35.4 (17.8, 57.2)	
Months from seroconversion to CHI-cART	48.1 (30.0, 69.5)	24.2 (13.0, 43.7)	24.9 (13.4, 45.1)	<0.001
Year of CHI-cART	2008 (2006, 2010)	2009 (2007, 2011)	2009 (2007, 2011)	<0.001
Follow-up after CHI-cART (months)	53.8 (30.2, 76.8)	33.9 (16.4, 58.3)	34.7 (16.6, 59.4)	<0.001
1st CD4^+^ after seroconversion (cells/μl)	540 (4512, 672)	486 (363, 637)	489 (367, 639)	<0.001
CHI-cART baseline CD4^+^ (cells/μl)	360 (276, 480)	329 (250, 428)	330 (251, 431)	<0.001
1st viral load after seroconversion (log_10_ copies/ml)	5.3 (4.7, 5.9)	4.7 (4.1, 5.3)	4.8 (4.1, 5.3)	<0.001
CHI-cART baseline viral load (log_10_ copies/ml)	4.8 (4.3, 5.2)	4.7 (4.2, 5.2)	4.7 (4.2, 5.2)	0.180

Numbers are *N* (%) or median (IQR). *P*-values based on (categorical variables) and Mann–Whitney (continuous variables) tests. cART, combined antiretroviral treatment; CHI chronic HIV infection; EHI, early HIV infection; IDU, injection drug users, IQR, interquartile range; MSM, men who have sex with men; MSW, sex between men and women; NNRTI, nonnucleoside reverse transcriptase inhibitor; PI, protease inhibitor.

Estimation of the seroconversion date among pretreated individuals was mostly accurate as 65.8% of them had a seroconversion illness, laboratory evidence of seroconversion or an HIV test interval of less than a month (Table 1). Among those with a longer HIV test interval (*n* = 69), the length of this interval was between 6 and 12 months for only 14 individuals. The median [interquartile range (IQR)] viral load at the start of their transient ART was 5.1 (4.5–5.8) log_10_ copies/ml. Out of 184 pretreated individuals with viral load measurements available during their EHI transient treatment, 133 (72%) had less than 50 and 29 (16%) 50–500 HIV-RNA copies/ml before interrupting treatment.

### Virologic response and relapse

Timing and frequency of viral load measurements after cART initiation in CHI were similar in the two groups, with the first measurement taken after a median (IQR) of 1.2 (0.9, 2.3) months.

**Fig. 1:**
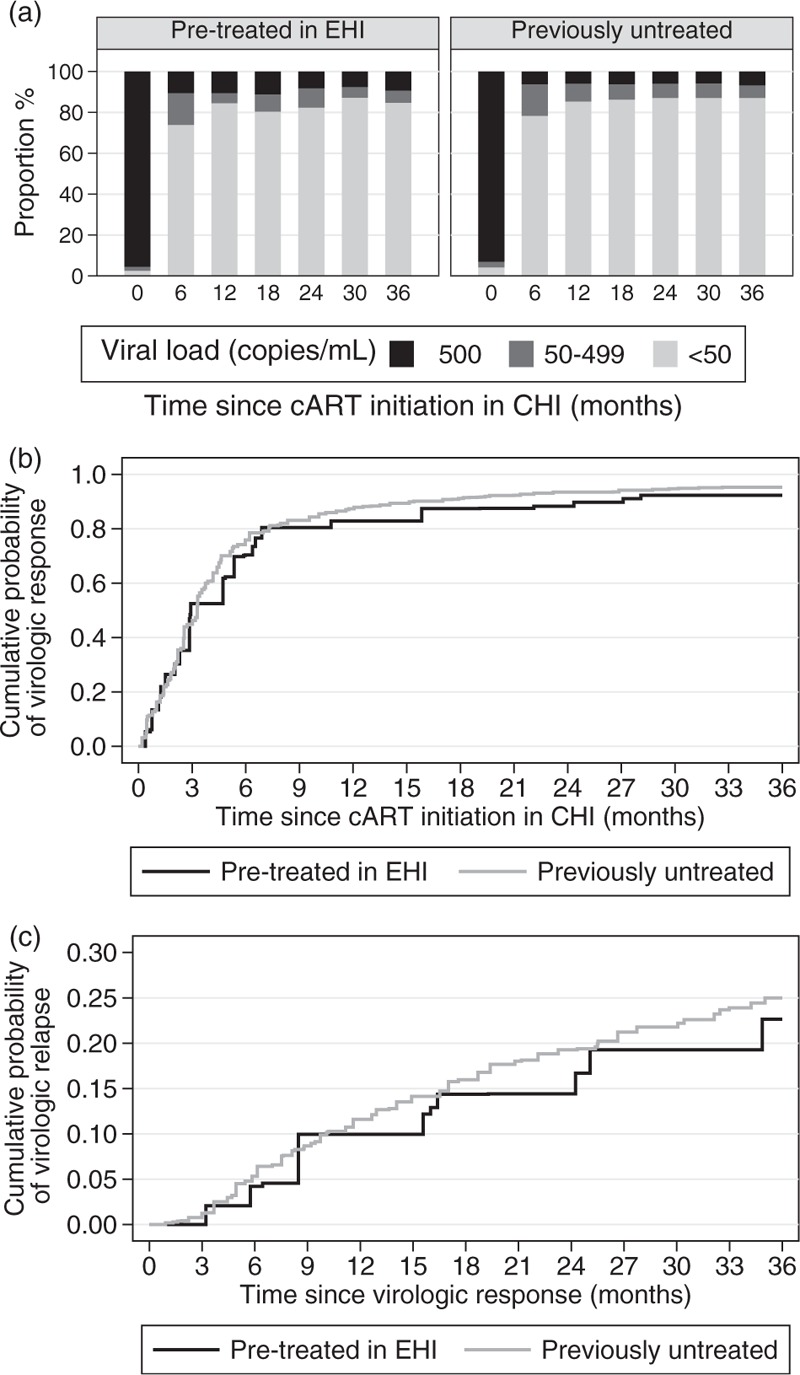
Cross-sectional distribution of (a) viral load, (b) virologic response and (c) virologic relapse after combination antiretroviral treatment initiation during chronic HIV infection (CHI) for individuals pretreated in early HIV infection (EHI) and previously untreated individuals.

The distribution of viral loads at different time points after CHI-cART initiation is summarized in Fig. 1a. As shown in this figure, more than 80% of the treated individuals in both groups achieved less than 50 copies/ml viral load levels after the 12th month of treatment. Although the proportion of individuals with less than 50 copies/ml viral load appears slightly higher at 6, 18, and 24 months after cART initiation in CHI in those who were previously untreated, compared to those pretreated in EHI, such figures should be interpreted with caution because of their cross-sectional nature.

The estimated cumulative probabilities of achieving less than 50 copies/ml viral load are presented in Fig. 1b. Median [95% confidence interval (CI)] time to virologic response was similar at 3.42 (2.82, 4.01) and 3.06 (2.94, 3.18) months for the pretreated in EHI and previously untreated group, respectively.

Multivariable analysis (Table 2) did not reveal any significant difference between the two groups (*P* = 0.585). Estimated acceleration factors were greater than 1 for all other risk groups compared to MSM, indicating longer average times to achieve viral suppression. A clear trend for higher rates of virologic response to treatment initiated in more recent years was also evident. Higher initial viral load levels were associated with longer times to virologic response. Finally, regimens based on NNRTIs were associated with better performance as the average times to achieve viral load undetectability were shorter compared with those associated with PI-based regimens.

**Table 2:** Differences in (a) virologic response and (b) virologic relapse of HIV-1 seroconverters treated in chronic infection by whether they had initiated combination antiretroviral treatment within 6 months of seroconversion (pretreated in early HIV infection) or not (not pretreated in early HIV infection).

	(a) Virologic response			(b) Virologic relapse		
Factor	Acceleration factors	95% CI	*P*	Acceleration factors	95% CI	*P*
Pretreated in early HIV infection (yes/no)	1.04	(0.90, 1.21)	0.585	1.27	(0.88, 1.85)	0.206
Sex (female/male)				0.63	(0.45, 0.88)	0.007
Risk group						
IDU/MSM	1.22	(1.02, 1.47)	0.034	0.33	(0.21, 0.54)	<0.001
MSW/MSM	1.09	(1.01, 1.19)	0.037	0.97	(0.73, 1.28)	0.82
Other unknown/MSM	1.28	(1.08, 1.52)	0.004	0.67	(0.42, 1.06)	0.087
Ethnic group						
Black/white	0.94	(0.76, 1.16)	0.578	1.08	(0.63, 1.88)	0.776
Hispanic/white	0.98	(0.65, 1.48)	0.926	1.28	(0.41, 3.97)	0.667
Asian/white	0.86	(0.63, 1.18)	0.354	0.71	(0.32, 1.60)	0.413
Mixed other/white	1.14	(0.73, 1.79)	0.569	0.86	(0.24, 3.00)	0.809
Unknown/white	1.09	(1.02, 1.17)	0.008	1.01	(0.84, 1.20)	0.935
Year of CHI cART						
2000−/2010+	1.66	(1.46, 1.89)	<0.001	0.32	(0.23, 0.44)	<0.001
2004−/2010+	1.35	(1.22, 1.51)	<0.001	0.47	(0.36, 0.63)	<0.001
2006−/2010+	1.21	(1.10, 1.32)	<0.001	0.63	(0.49, 0.80)	<0.001
2008−/2010+	1.11	(1.02, 1.20)	0.011	0.71	(0.57, 0.88)	0.002
Type of CHI cART						
Boosted PI/NNRTI	1.34	(1.25, 1.43)	<0.001	0.77	(0.65, 0.91)	0.003
Unboosted PI/NNRTI	1.24	(1.03, 1.50)	0.023	0.74	(0.44, 1.23)	0.246
Other/NNRTI	0.53	(0.45, 0.62)	<0.001	0.79	(0.56, 1.12)	0.19
CHI cART baseline RNA (copies/ml)						
500−/50–499	0.87	(0.66, 1.14)	0.315	1.94	(1.27, 2.97)	0.002
5000−/50–499	1.91	(1.50, 2.44)	<0.001	2.57	(1.83, 3.60)	<0.001
50000−/50–499	3.09	(2.41, 3.96)	<0.001	1.81	(1.26, 2.61)	0.001
100000+/50–499	4.14	(3.25, 5.28)	<0.001	1.82	(1.29, 2.56)	0.001
Age at CHI-cART (per 10 years)				1.15	(1.06, 1.25)	0.001

*P* values based on Wald tests from the corresponding models. Results from accelerated failure time models for interval censored data. cART, combination antiretroviral treatment; CHI, chronic infection; CI, confidence interval; IDU, injection drug users, IQR, interquartile range; MSM, men who have sex with men; MSW, sex between men and women; NNRTI, nonnucleoside reverse transcriptase inhibitor; PI, protease inhibitor.

Approximately 20% of those who achieved virologic suppression (or initiated cART in CHI with undetectable viral load) relapsed by the 24th month after the initial virologic response (Fig. 1c). The estimated cumulative probabilities (95% CI) of virologic relapse were similar for the two groups at 0.09 (0.05, 0.12) and 0.11 (0.10, 0.12) at the 12th month, and 0.16 (0.12, 0.21) and 0.20 (0.18, 0.21) at the 24th month after the initial response for those pretreated in EHI and those previously untreated, respectively. After adjusting for other potential confounders (Table 2) differences between the two groups remained nonsignificant (*P* = 0.206). Relapse rates were higher in men, injecting drug users, those treated in earlier years and those (re)starting treatment with very low viral load.

We also found no evidence to suggest that virologic response or relapse following reinitiation in CHI were associated with the interval between estimated seroconversion and cART initiation in EHI (*P* = 0.624 and *P* = 0.581, respectively). Similarly, there was no evidence of an effect of the cART duration during EHI (*P* = 0.866 and *P* = 0.844, respectively).

### CD4^+^ cell count evolution

The first CD4^+^ cell count measurement post CHI-cART initiation was taken after a median (IQR) of 1.3 (0.9, 2.5) months with no significant difference between the two groups.

**Fig. 2:**
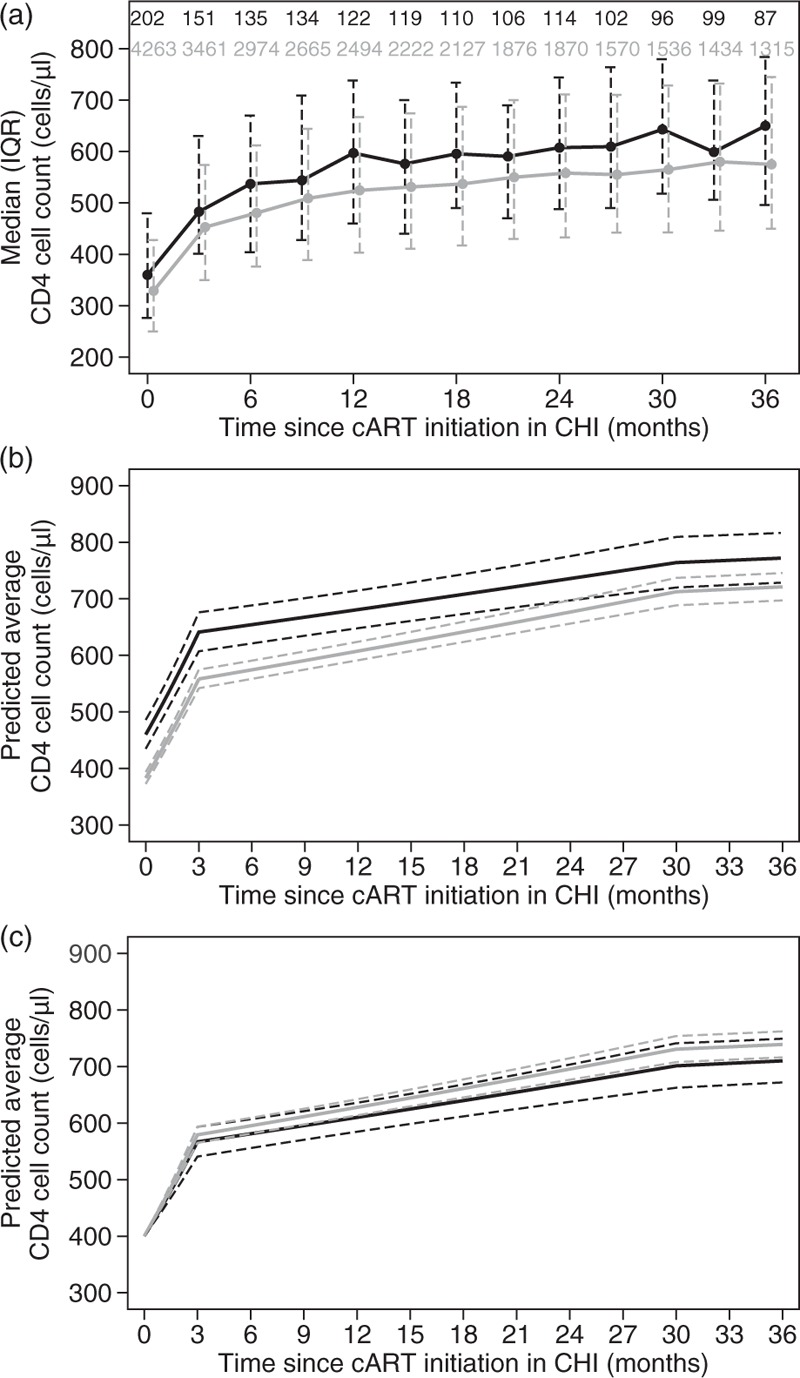
Cross-sectional distribution of (a) CD4^+^ cell count, (b) estimated evolution of average CD4^+^ cell count and (c) estimated evolution of average CD4^+^ cell count assuming a common baseline of 400 cells/μl after combination antiretroviral treatment initiation during chronic HIV infection (CHI) in individuals pretreated (black lines) in early HIV infection (EHI) and previously untreated (grey lines) individuals.

The distribution of CD4^+^ cell count by time and group is summarized in Fig. 2a. Individuals who had initiated cART in EHI seem to start their CHI-cART at slightly higher CD4^+^ cell counts. CD4^+^ cell count increases seem faster during the first 3 months of therapy with decelerating rates thereafter. CD4^+^ dynamics seem similar between the two groups with no marked differences, in terms of CD4^+^ cell count slope but, as in Fig. 1a, caution is needed when interpreting such cross-sectional figures.

Vertical lines in (a) denote interquartile range (IQR) and dashed lines in (b) and (c) are 95% confidence intervals.

Results from a multivariable mixed model are shown in Table 3. Those pretreated in EHI tend to start their CHI treatment at higher CD4^+^ cell counts compared to those who were previously naive. The estimated average (95% CI) baseline CD4^+^ cell counts for the pretreated and previously naive individuals, for the reference category (white, MSM, aged 15–29 years at seroconversion, starting an NNRTI-based regimen after 2009, with >100 000 HIV-RNA copies/ml), were 460 (434, 486) and 383 (373, 394) cells/μl, respectively (*P* < 0.001). Individuals initiating in CHI had faster CD4^+^ increases in general, but the difference was significant only during months 3–30 after treatment initiation (*P* = 0.015). Boosted PI-based regimens were associated with faster increases during the first 3 months and injecting drug users had a worse CD4^+^ response, compared to men infected through sex between men throughout the whole study period. Younger individuals had faster initial CD4^+^ cell count increases but this association was reversed after the first 3 months of treatment. Finally, individuals of black or Hispanic ethnicity had lower CD4^+^ cell counts at baseline, compared with white individuals, whereas higher baseline plasma viral load levels were associated with lower baseline CD4^+^ cell count but faster increases after treatment initiation.

**Table 3:** CD4^+^ cell count evolution after combination antiretroviral treatment initiation during chronic infection.

	CD4^+^ cell count at CHI cART initiation (square root cells/μl)			Rate of CD4^+^ cell count change (0–3 months after CHI cART initiation) (square root cells/μl per month)			Rate of CD4^+^ cell count change (3–30 months after CHI cART initiation) (square root cells/μl per year)			Rate of CD4^+^ cell count change (30+ months after CHI cART initiation) (square root cells/μl per year)		
Factor	Estimate	95% CI	*P*	Estimate	95% CI	*P*	Estimate	95% CI	*P*	Estimate	95% CI	*P*
Reference category	19.57	(19.30, 19.84)	<0.001	1.35	(1.25, 1.45)	<0.001	1.36	(1.18, 1.55)	<0.001	0.32	(0.21, 0.44)	<0.001
Pretreated in early HIV infection/previously untreated	1.87	(1.31, 2.43)	<0.001	−0.06	(−0.21, 0.10)	0.456	−0.33	(−0.60, −0.06)	0.015	−0.04	(−0.23, 0.16)	0.714
Type of CHI cART												
Boosted PI/NNRTI				0.12	(0.06, 0.18)	<0.001						
Unboosted PI/NNRTI				0.08	(−0.10, 0.25)	0.399						
Other/NNRTI				0.22	(0.10, 0.34)	<0.001						
Year of CHI cART												
2000–2003/2010+	−1.56	(−2.05, −1.06)	<0.001	−0.02	(−0.15, 0.12)	0.777	−0.75	(−0.98, −0.52)	<0.001			
2004–2005/2010+	−2.98	(−3.39, −2.57)	<0.001	0.09	(−0.02, 0.20)	0.098	−0.17	(−0.37, 0.03)	0.096			
2006–2007/2010+	−2.85	(−3.20, −2.51)	<0.001	0.18	(0.09, 0.28)	<0.001	−0.12	(−0.30, 0.05)	0.175			
2008–2009/2010+	−1.65	(−1.94, −1.36)	<0.001	0.16	(0.08, 0.24)	<0.001	−0.17	(−0.33, −0.01)	0.035			
Risk group												
IDU/MSM	−1.67	(−2.32, −1.03)	<0.001				−0.50	(−0.83, −0.17)	0.003	−0.47	(−0.74, −0.21)	<0.001
MSW/MSM	−0.36	(−0.66, −0.07)	0.016				−0.03	(−0.18, 0.12)	0.716	0.02	(−0.11, 0.14)	0.776
Other unknown/MSM	−0.31	(−0.93, 0.31)	0.324				−0.13	(−0.46, 0.20)	0.443	0.06	(−0.28, 0.39)	0.749
Age at CHI cART initiation												
30–39/15–29				−0.11	(−0.19, −0.03)	0.005	0.29	(0.13, 0.45)	<0.001	0.09	(−0.04, 0.23)	0.184
40–49/15–29				−0.13	(−0.22, −0.04)	0.003	0.22	(0.05, 0.40)	0.013	0.18	(0.04, 0.33)	0.015
50+/15–29				−0.16	(−0.26, −0.05)	0.004	0.23	(0.01, 0.44)	0.036	−0.04	(−0.22, 0.13)	0.624
CHI cART baseline RNA (copies/ml)												
<500/100 000+	3.71	(3.22, 4.20)	<0.001	−1.00	(−1.14, −0.86)	<0.001	−0.49	(−0.75, −0.22)	<0.001			
500–4999/100 000+	1.85	(1.40, 2.30)	<0.001	−0.63	(−0.75, −0.50)	<0.001	−0.45	(−0.69, −0.20)	<0.001			
5000–49 999/100 000+	0.70	(0.42, 0.98)	<0.001	−0.33	(−0.41, −0.26)	<0.001	−0.09	(−0.24, 0.05)	0.195			
50 000–99 999/100 000+	0.26	(−0.08, 0.60)	0.136	−0.24	(−0.33, −0.15)	<0.001	0.06	(−0.11, 0.23)	0.470			
Ethnic												
Black/white	−0.96	(−1.72, −0.20)	0.013	0.02	(−0.18, 0.22)	0.853						
Hispanic/white	−1.56	(−3.04, −0.08)	0.038	0.14	(−0.27, 0.54)	0.501						
Asian/white	−0.10	(−1.25, 1.05)	0.863	−0.19	(−0.50, 0.13)	0.244						
Mixed/other/white	−1.14	(−2.80, 0.53)	0.180	0.25	(−0.19, 0.70)	0.266						
Unknown/white	0.05	(−0.19, 0.29)	0.670	−0.08	(−0.15, −0.02)	0.016						
AIDS/AIDS free (before cART at CHI)	−1.27	(−1.85, −0.69)	<0.001				0.37	(0.10, 0.65)	0.008			

*P* values based on Wald tests from the corresponding models. Results from a multivariable piecewise linear mixed model. cART, combination antiretroviral treatment; CHI, chronic infection; CI, confidence interval; IDU, injection drug users, IQR, interquartile range; MSM, men who have sex with men; MSW, sex between men and women; NNRTI, nonnucleoside reverse transcriptase inhibitor; PI, protease inhibitor.

The time interval between seroconversion and initiation of treatment in EHI for the pretreated group did not significantly affect the initial CD4^+^ cell count at treatment initiation in CHI or the subsequent rates of CD4^+^ cell count increase (*P* = 0.722). The duration of the transient EHI treatment was positively associated with the baseline CD4^+^ cell counts at CHI treatment initiation (*P* = 0.020) but had no significant effect on the subsequent CD4^+^ slopes (*P* > 0.100 for all three slopes).

The average CD4^+^ cell count evolution for the reference category (as previously described) is shown in Fig. 2b. Those initiating in CHI had slightly faster CD4^+^ cell count increases, compared to those who had initiated in EHI, but their CD4^+^ cell levels remain lower, even after 3 years of treatment (estimated difference: 51 CD4^+^ cells/μl; *P* = 0.007). However, as pretreated individuals restart treatment in CHI at higher CD4^+^ cell count compared to those who were initiating in CHI, we applied a postestimation adjustment assuming individuals in both groups re(initiate) treatment at the same CD4^+^ cell count level (i.e., 400 CD4^+^ cells/μl). As can be seen in Fig. 2c, those initiating in CHI seem to maintain slightly higher CD4^+^ cell counts compared with those pretreated during EHI, but there is a large degree of overlap in the corresponding 95% CIs, indicating that differences are not significant (*P* = 0.077).

## Discussion

Our study assessed virologic and immunologic outcomes of long-term cART, initiated during CHI, in persons who were pretreated with transient cART during EHI compared with those who initiated in CHI. Our findings suggest that prior treatment during EHI has no effect on virologic response or relapse following reinitiation of long-term cART. Pretreated individuals reinitiated cART in CHI at approximately 80 cells/μl higher CD4^+^ cell count than those initiating in CHI. Even though the subsequent rate of CD4^+^ cell count increase was slightly slower for those who had previously been treated in EHI; they retained higher CD4^+^ cell counts even after 3 years of treatment. However, assuming a common baseline CD4^+^ cell count, the estimated difference between the two groups was not significant after 3 years of treatment. Duration of transient cART in EHI and its timing relative to estimated seroconversion did not appear to have any significant effect on virologic or immunologic outcome of long-term cART in CHI. We cannot rule out, however, that shorter cART duration in EHI would have adversely affected outcome following reinitiation in CHI.

The main strength of our study is its size and length of follow-up. To our knowledge, this is the largest study focusing on this comparison, including 202 pretreated and 4263 previously untreated individuals with a median follow-up, after the initiation of treatment in CHI, of approximately 4.5 and 3 years, respectively. Given that this is an observational study, efforts have been made to minimise the effect of bias. For example, we excluded individuals who received cART in EHI because of low CD4^+^ cell counts or AIDS. Moreover, all comparisons were based on multivariable models which included adjustments for potential confounders. Finally, the study population is drawn from a number of countries (mainly in Europe, but also Australia and Canada), and includes individuals who had acquired HIV through both sex between men, and sex between men and women, and regimens used for long-term cART were equally based on the two most common antiretroviral classes (i.e., NNRTIs and boosted PIs).

Despite our restrictions regarding the time interval between the last negative and first-positive HIV test dates, a substantial proportion of individuals (mainly among those who initiated cART during chronic infection) had an HIV test window between 30 days and 1 year, allowing for some uncertainty regarding the exact time of seroconversion. It is noteworthy though that, for almost two-thirds of the pretreated individuals, the accuracy in the estimation of the seroconversion date was excellent and most of them initiated their transient treatment very soon after their seroconversion, having viral loads which were much higher compared to typical set-point levels. Thus, although we did not specifically aim to study individuals treated during acute infection, a substantial proportion of the pretreated individuals started cART very close to the time of seroconversion and this allowed us to investigate whether the gap between seroconversion and ART initiation modified our findings.

Additionally, reasons for starting and stopping treatment in EHI were not available. It should be noted though that the frequency of seroconversion illnesses was low, and similar in both groups, and that those who started treatment in EHI because of an AIDS diagnosis and/or low CD4^+^ cell counts have been excluded. Furthermore, virological failure does not appear to account for the interruption of early treatment as almost 90% of pretreated individuals interrupted with HIV-RNA less than 500 copies/ml.

Even though differences in available factors with a significant effect on virologic and/or immunologic response were taken into account, residual confounding cannot be ruled out, as in all observational studies. Moreover, we were not able to examine differences between groups in terms of proinflammation and coagulation markers, HIV reservoir size, or CD4^+^/CD8^+^ ratio normalization, thus our findings should be interpreted with caution.

Our main results are in agreement with those reported by the Short Pulse Anti Retroviral Therapy at HIV Seroconversion trial investigators [16]. As in our study, individuals randomized to 12 or 48 weeks of transient cART within 6 months from seroconversion had similar virologic and CD4^+^ responses in long-term cART initiated in CHI with those who remained untreated during EHI. In a smaller study, Grijsen and colleagues [32] compared 36 ART-naive and 132 individuals who were pretreated in EHI and found no differences between them in terms of virologic and CD4^+^ cell count response. Notably, in the same study it was found that transient treatment in EHI was not associated with clinically relevant drug resistance mutations.

Guidelines regarding the optimal clinical management of EHI were for many years, and partly remain, unclear [26–28]. However, there is accumulated evidence supporting the hypothesis that intervening as soon as possible after HIV infection may lead to clinical benefit or even remission of the disease in a small number of cases [6–9,13,23,24,33]. Despite these encouraging findings, the proportion of early treated individuals who maintain long-term viral suppression after treatment cessation is very small. A recent meta-analysis [34] summarized the results of eight relevant studies and found that ‘immunovirological benefits declined gradually after treatment interruption, reaching no statistical significance after 12–24 months’.

As HIV treatment guidelines have successively recommended cART initiation at higher CD4^+^ cell count thresholds [26–28], and the latest evidence from the INISGHT-START trial [35] clearly supports the initiation of treatment in all HIV-positive individuals, irrespectively of their CD4^+^ cell count, a significant number of individuals will likely be initiating cART in EHI. We may anticipate a substantial proportion of them to interrupt, who will need to reinitiate later during chronic infection. Thus, it is clinically relevant to assess the effects of this early intervention on the outcomes of long-term treatment. According to our findings, individuals who initiate, and then stop, cART within 6 months of seroconversion do not fare differently from those who initiate in CHI, at least in terms of viral load and CD4^+^ cell count post-cART changes.

Treatment interruption during CHI is associated with poorer prognosis [5,36] and clearly not recommended by current guidelines [26,27]. Our data suggest, in agreement with others, that interrupting cART initiated in EHI does not have a detrimental effect on the immunovirologic response to long-term treatment in CHI. Intervening during EHI to suppress viral load, during a period which is characterized by markedly higher risk of HIV transmission, could have a positive impact in terms of public health [37]. Without evidence regarding the safety and consequences of interrupting treatment initiated in EHI and restarting it later, this strategy should be avoided. Our findings, nonetheless, provide helpful insights for those designing cure studies which necessitate stopping cART initiated in EHI to assess the degree of viral remission.

## Acknowledgements

Funding: The research leading to these results has received funding from the European Union Seventh Framework Programme (FP7/2007–2013) under EuroCoord grant agreement n° 260694. K.P. is funded through a grant from the UK Medical Research Council (MC_UU_12023/1 – Infections).

Authors’ contributions: Conceived and designed the experiments: N.P., G.T., and K.P. Analyzed the data: N.P. Wrote the article: N.P., G.T., and K.P. Drafted manuscript with critical revision for important intellectual content undertaken: L.M., A.O., D.C., A.D.K., I.L., M.L.C., M.F., and S.M.

### Conflicts of interest

There are no conflicts of interest.

## Supplementary Material

Supplemental Digital Content
