# Supplementary material for: The impact of transient combination antiretroviral treatment in early HIV infection on viral suppression and immunologic response in later treatment
Source: AIDS. 2016 Mar 7;30(6):879–88. doi: 10.1097/QAD.0000000000000991 (PMC4794189; doi:10.1097/QAD.0000000000000991)
Supplement: Supplemental Digital Content [file aids-30-879-s001.pdf]

**Figure S1. Flow-chart for the selection of the study population.**
(EHI: Early HIV Infection; CHI: Chronic HIV Infection)

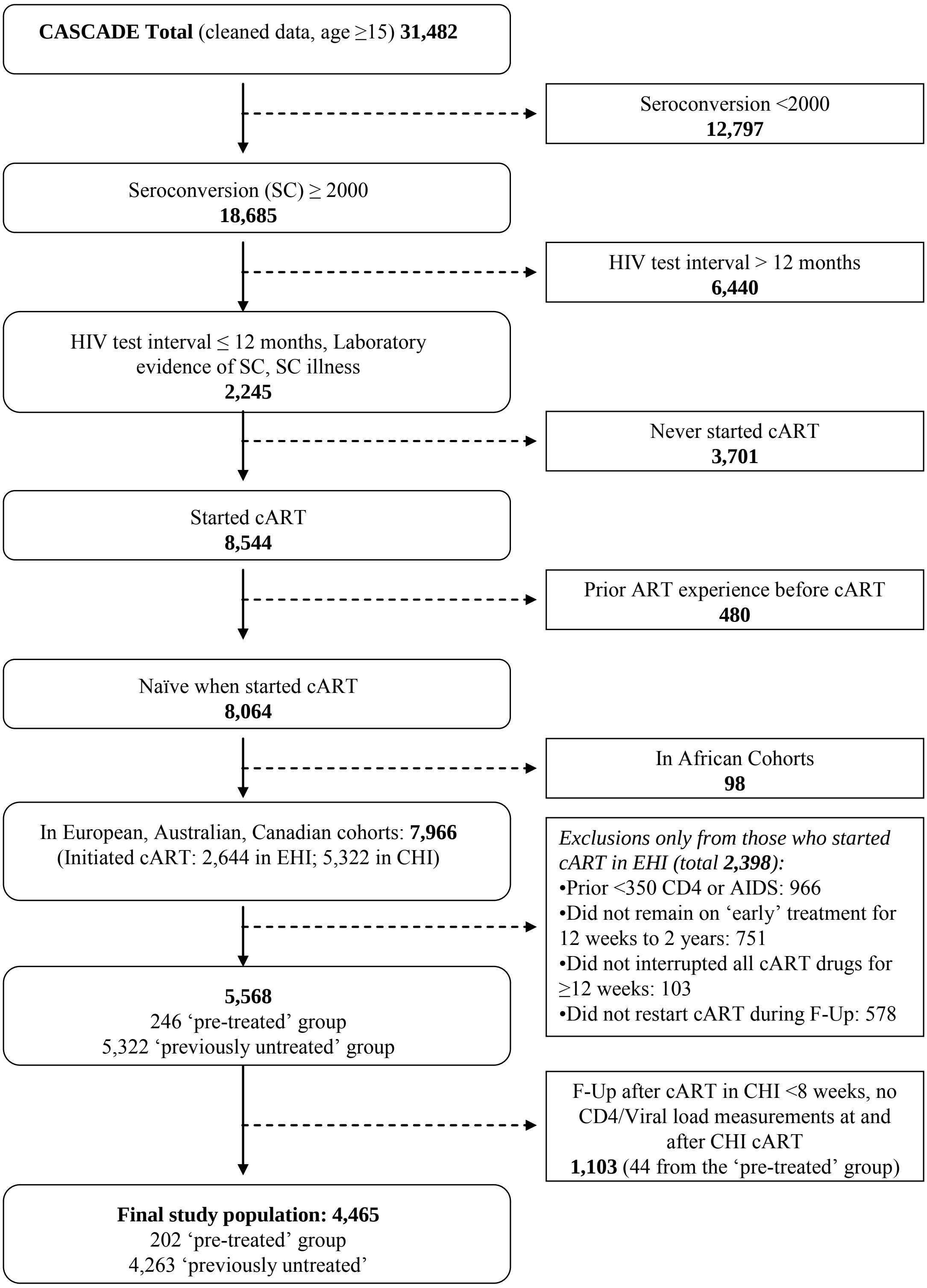

**APPENDIX**

**CASCADE Steering Committee**: Julia Del Amo (Chair), Laurence Meyer (Vice Chair), Heiner C. Bucher, Geneviève Chêne, Osamah Hamouda, Deenan Pillay, Maria Prins, Magda Rosinska, Caroline Sabin, Giota Touloumi.

**CASCADE Co-ordinating Centre:** Kholoud Porter (Project Leader), Ashley Olson, Andrea Cartier, Lorraine Fradette, Sarah Walker, Abdel Babiker.

**CASCADE Clinical Advisory Board:** Heiner C. Bucher, Andrea De Luca, Martin Fisher, Roberto Muga

**CASCADE Collaborators: Australia** PHAEDRA cohort (Tony Kelleher, David Cooper, Robert Finlayson, Mark Bloch) Sydney AIDS Prospective Study and Sydney Primary HIV Infection cohort (Tony Kelleher, Tim Ramacciotti, Linda Gelgor, David Cooper, Don Smith); **Austria** Austrian HIV Cohort Study (Robert Zangerle); **Canada** South Alberta clinic (John Gill); **Estonia** Tartu Ülikool (Irja Lutsar); **France** ANRS CO3 Aquitaine cohort (Linda Wittkop, Francois Dabis, Rodolphe Thiebaut), ANRS CO4 French Hospital Database (Dominique Costagliola, Marguerite Guiguet), Lyon Primary Infection cohort (Philippe Vanhems), French ANRS CO6 PRIMO cohort (Marie-Laure Chaix, Jade Ghosn), ANRS CO2 SEROCO cohort (Laurence Meyer, Faroudy Boufassa); **Germany** German HIV-1 seroconverter cohort (Osamah Hamouda, Claudia Kücherer, Barbara Bartmeyer); **Greece** AMACS (Helen Sambatakou, Nikolaos V Sipsas, Charalambos A Gogos); Greek Haemophilia cohort (Giota Touloumi, Nikos Pantazis, Olga Katsarou); **Italy** Italian Seroconversion Study (Giovanni Rezza, Maria Dorrucci), ICONA cohort (Antonella d'Arminio Monforte, Andrea De Luca.) **Netherlands** Amsterdam Cohort Studies among homosexual men and drug users (Maria Prins, Ronald Geskus, Jannie van der Helm, Hanneke Schuitemaker); **Norway** Oslo and Ulleval Hospital cohorts (Mette Sannes, Oddbjorn Brubakk, Anne-Marte Bakken Kran, Anne Margarita Dyrhol Riise); **Poland** National Institute of Hygiene (Magdalena Rosinska); **Spain** Badalona IDU hospital cohort (Roberto Muga, Jordi Tor), Barcelona IDU Cohort (Patricia Garcia de Olalla, Joan Cayla), CoRIS-scv (Julia del Amo, Santiago Moreno, Susana Monge); Madrid cohort (Julia Del Amo, Jorge del Romero), Valencia IDU cohort (Santiago Pérez-Hoyos); **Sweden** Swedish InfCare HIV Cohort, Sweden (Anders Sönnerborg); **Switzerland** Swiss HIV Cohort Study (Heiner C. Bucher, Huldrych Günthard, Martin Rickenbach); **Ukraine** Perinatal Prevention of AIDS Initiative (Ruslan Malyuta); **United Kingdom** Public Health England (Gary Murphy), UK Register of HIV Seroconverters (Kholoud Porter, Anne Johnson, Andrew Phillips, Abdel Babiker), University College London (Deenan Pillay); **African cohorts:** Genital Shedding Study (US: Charles Morrison; Family Health International, Robert Salata, Case Western Reserve University, Uganda: Roy Mugerwa, Makerere University, Zimbabwe: Tsungai Chipato, University of Zimbabwe); International AIDS Vaccine Initiative (IAVI) Early Infections Cohort (Kenya, Rwanda, South Africa, Uganda, Zambia: Pauli N. Amornkul, IAVI, USA; Jill Gilmour, IAVI, UK; Anatoli Kamali, Uganda Virus Research Institute/Medical Research Council Uganda; Etienne Karita, Projet San Francisco, Rwanda).

**EuroCoord Executive Board**: Fiona Burns, University College London, UK; Geneviève Chêne (Chair), University of Bordeaux, France; Dominique Costagliola (Scientific Coordinator), Institut National de la Santé et de la Recherche Médicale, France; Carlo Giaquinto, Fondazione PENTA, Italy; Jesper Grarup, Region Hovedstaden, Denmark; Ole Kirk, Region Hovedstaden, Denmark; Laurence Meyer, Institut National de la Santé et de la Recherche Médicale, France; Heather Bailey, University College London, UK; Alain Volny Anne, European AIDS Treatment Group, France; Alex Panteleev, St. Petersburg City AIDS Centre, Russian Federation; Andrew Phillips, University College London, UK, Kholoud Porter, University College London, UK; Claire Thorne, University College London, UK.

**EuroCoord Council of Partners**: Jean-Pierre Aboulker, Institut National de la Santé et de la Recherche Médicale, France; Jan Albert, Karolinska Institute, Sweden; Silvia Asandi, Romanian Angel Appeal Foundation, Romania; Geneviève Chêne, University of Bordeaux, France; Dominique Costagliola, INSERM, France; Antonella d'Arminio Monforte, ICoNA Foundation, Italy; Stéphane De Wit, St. Pierre University Hospital, Belgium; Peter Reiss, Stichting HIV Monitoring, Netherlands; Julia Del Amo, Instituto de Salud Carlos III, Spain; José Gatell, Fundació Privada Clínic per a la Recerca Bíomèdica, Spain; Carlo Giaquinto, Fondazione PENTA, Italy; Osamah Hamouda, Robert Koch Institut, Germany; Igor Karpov, University of Minsk, Belarus; Bruno Ledergerber, University of Zurich, Switzerland; Jens Lundgren, Region Hovedstaden, Denmark; Ruslan Malyuta (Chair), Perinatal Prevention of AIDS Initiative, Ukraine; Claus Møller, Cadpeople A/S, Denmark; Kholoud Porter, University College London, United Kingdom; Maria Prins, Academic Medical Centre, Netherlands; Aza Rakhmanova, St. Petersburg City AIDS Centre, Russian Federation; Jürgen Rockstroh, University of Bonn, Germany; Magda Rosinska, National Institute of Public Health, National Institute of Hygiene, Poland; Manjinder Sandhu, Genome Research Limited; Claire Thorne, University College London, UK; Giota Touloumi, National and Kapodistrian University of Athens, Greece; Alain Volny Anne, European AIDS Treatment Group, France.

**EuroCoord External Advisory Board**: David Cooper, University of New South Wales, Australia; Nikos Dedes, Positive Voice, Greece; Kevin Fenton, Public Health England, USA; David Pizzuti, Gilead Sciences, USA; Marco Vitoria, World Health Organisation, Switzerland.

**EuroCoord Secretariat:** Silvia Faggion, Fondazione PENTA, Italy; Lorraine Fradette, University College London, UK; Richard Frost, University College London, UK; Andrea Cartier, University College London, UK; Dorthe Raben, Region Hovedstaden, Denmark; Christine Schwimmer, University of Bordeaux, France; Martin Scott, UCL European Research & Innovation Office, UK.
